# Bridge Digital Twinning Using an Output-Only Bayesian Model Updating Method and Recorded Seismic Measurements

**DOI:** 10.3390/s22031278

**Published:** 2022-02-08

**Authors:** Farid Ghahari, Niloofar Malekghaini, Hamed Ebrahimian, Ertugrul Taciroglu

**Affiliations:** 1 Department of Civil & Environmental Engineering, University of California, Los Angeles, CA 90095, USA; ghahari@seas.ucla.edu (F.G.); etacir@ucla.edu (E.T.); 2 Department of Civil & Environmental Engineering, University of Nevada, Reno, NV 89557, USA; nmalekghaini@nevada.unr.edu

**Keywords:** structural health monitoring, digital twin, damage diagnosis, finite element model updating, Bayesian inference, soil-structure interaction, foundation input motion, rapid post-earthquake assessment

## Abstract

Rapid post-earthquake damage diagnosis of bridges can guide decision-making for emergency response management and recovery. This can be facilitated using digital technologies to remove the barriers of manual post-event inspections. Prior mechanics-based Finite Element (FE) models can be used for post-event response simulation using the measured ground motions at nearby stations; however, the damage assessment outcomes would suffer from uncertainties in structural and soil material properties, input excitations, etc. For instrumented bridges, these uncertainties can be reduced by integrating sensory data with prior models through a model updating approach. This study presents a sequential Bayesian model updating technique, through which a linear/nonlinear FE model, including soil-structure interaction effects, and the foundation input motions are jointly identified from measured acceleration responses. The efficacy of the presented model updating technique is first examined through a numerical verification study. Then, seismic data recorded from the San Rogue Canyon Bridge in California are used for a real-world case study. Comparison between the free-field and the foundation input motions reveals valuable information regarding the soil-structure interaction effects at the bridge site. Moreover, the reasonable agreement between the recorded and estimated bridge responses shows the potentials of the presented model updating technique for real-world applications. The described process is a practice of digital twinning and the updated FE model is considered as the digital twin of the bridge and can be used to analyze the bridge and monitor the structural response at element, section, and fiber levels to diagnose the location and severity of any potential damage mechanism.

## 1. Introduction

The field of computational structural mechanics has advanced to a mature level to facilitate high-fidelity and computationally-efficient seismic response simulation of bridges [1,2,3]. Practitioners and researchers use mechanics-based Finite Element (FE) models for response prediction of complex bridge structures. Nevertheless, these models include inherent uncertainties when it comes to mirroring real-world response behavior. These can include uncertainties in soil and structural material models and parameters, dynamic input excitations, uncertainties in damping energy dissipation mechanisms, etc. The uncertainties can be quantified and/or reduced by integrating mechanics-based FE models with measured responses of the structure through a data assimilation approach. This approach consists of training/updating models with measurements to estimate uncertain/unknown model parameters [4] (i.e., input-output model updating) or uncertain/unknown model and input parameters [5,6] (i.e., output-only model updating). The trained/updated model provides a digital replicate of the real-world asset given the training data and is referred to as the Digital Twin (DT). Indeed, the DT can absorb new data (e.g., responses to future earthquakes) as they become available, thus offering an evolving and live platform that can be used for response prediction, asset management, rapid post-earthquake damage assessment, and decision-making for maintenance/rehabilitation of bridge infrastructures. Advancing towards this idea is the main motivation behind this research work.

Materializing the DT platform for bridges requires overcoming several technical hurdles. This paper is focused on the following challenges:

*(i)*  Nonlinearity in bridge response behavior: Several approaches for linear FE model updating using modal properties, identified from ambient and/or operational data, exist in the literature (e.g., [6,7,8,9,10]). Nevertheless, these methods can update the bridge model only in the linear regime of response. Linear FE model updating methods provide limited insight into the nonlinear response behavior of the structure, which can occur during strong earthquake events.
*(ii)*  Measurement sparsity: Through the California Strong Motion Instrumentation Program (CSMIP) [11], the California Department of Conservation in collaboration with the California Department of Transportation (Caltrans) has instrumented several bridges and recorded their seismic responses during the past three decades. This valuable dataset has benefited the research community [12,13,14] and can provide the baseline for developing a DT platform for instrumented bridges across California. However, the collected data is often subjected to notable instrumentation sparsity. The sparsity in data poses important challenges to the uniqueness of the solution for the model updating technique and the accuracy of the resulting DT.
*(iii)*  Soil-Structure Interaction (SSI): The Foundation Input Motions (FIMs), which are the theoretical inputs to the soil-structure interactive system, are not explicitly measurable. FIMs can be different from the Free-Filed Motions (FFMs) due to the SSI effects [15]. The available knowledge on SSI effects is limited to analytical and numerical studies, and the in-situ and real-world effects of SSI on complex structures are not completely known [16,17,18,19,20]. Hence, developing DT for bridges using seismic measurements may require the estimation of the FIMs.

In this paper, to transcend the aforementioned technical challenges in the application of digital twinning and virtual sensing, an output-only model updating technique in the time domain is presented. Through this presented technique, the uncertain parameters of a linear or nonlinear mechanics-based FE model along with the FIMs can be estimated using sparsely measured acceleration responses recorded during an earthquake. The model updating technique presented herein is mainly based on a sequential Bayesian inference algorithm originally developed in previous works [5,21]. The efficacy of the presented model updating technique is verified through numerically simulated data using the San Roque Canyon (SRC) bridge model as a testbed. Then, the seismic data collected from the SRC bridge are used for real-world case studies. Although, the amplitudes of the available recorded motions are low, and thus the bridge mainly behaves in the linear-elastic regime, the process can be readily applied to strong earthquakes and nonlinear structural behavior. The main novelty of this paper is the application of the time-domain output-only model updating technique in real-world settings to investigate its efficacy and limitations.

The outline of the paper is as follows. First, the sequential Bayesian inference for output-only model updating technique and the identifiability analysis approach to determine the identifiable model parameters are briefly presented in Section 2. Section 3 is focused on the verification of the presented model updating technique using numerically simulated data. The FE model of SRC bridge is presented in detail in Section 3.1, and the identifiability analysis is performed in Section 3.2. Following that, the results of the verification study in a numerically simulated environment are presented in Section 3.3. Five case studies are carried out in Section 4 using real-world earthquake datasets and the results are discussed. In Section 5, the application of DT and virtual sensing is presented. Finally, the conclusion and future steps are discussed.

## 2. Sequential Bayesian Inference Method and Identifiability Analysis

The model updating technique for joint system and input estimation is based on a sequential Bayesian inference method using the unscented transformation approach [22] for uncertainty propagation. This technique will be reviewed briefly in Section 2.1. The Identifiability analysis process to quantify the information content of the measurement data and potential identifiability of model parameters is presented in Section 2.2.

### 2.1. Sequential Bayesian Inference Method for Output-Only FE Model Updating

**Figure 1:**
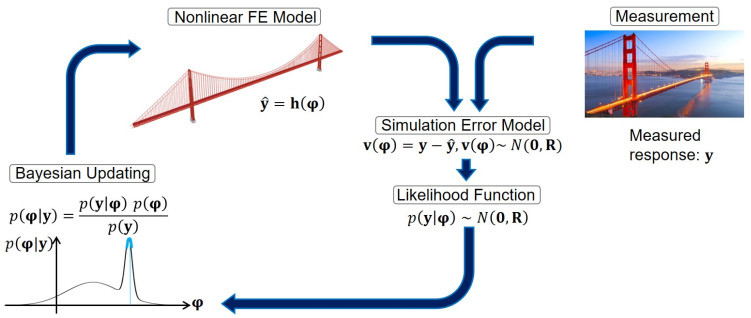
Schematic representation of the sequential Bayesian inference method for model updating.

The model updating technique for joint system and input estimation is schematically shown in Figure 1. This technique is closely similar to the works presented in [5,21], with some tweaks and improvements as will be outlined here. As shown in Figure 1, the unknown model parameters and inputs (here FIMs) are augmented into the unknown parameter vector **φ**, the uncertainties of which are expressed with a Gaussian Probability Density Function (PDF). These uncertainties are propagated into the FE model $\hat{y}=h\left(\varphi \right)$, in which $h\left(\ldots \right)$ is the nonlinear response function of the FE model. Next, a simulation (or prediction) error model $v\left(\varphi \right)\;$is defined to correlate the FE-predicted response ($\hat{y}$) with the measured response (y) collected by the sensors. Finally, the Bayes’ theorem is used to find the posterior PDF of the unknown parameters, which is then used as the prior PDF for the next sequence of measured responses.

**Figure 2:**
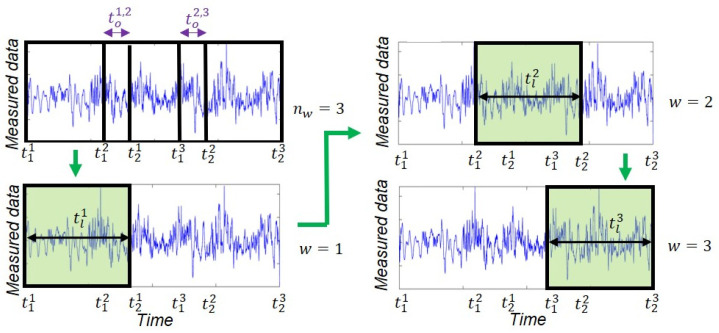
Schematic representation of the sequential estimation window approach. The estimation problem is completed at each estimation window iteratively and moves sequentially to the next estimation window.

A sequential estimation window approach is used in this study to improve the efficacy of the estimation process. In this approach, the time domain is divided into $n_{w}\geq 2$ successive overlapping estimation windows. The $w^{th}$ estimation window spans from time step $t_{1}^{w}$ to time step $t_{2}^{w}$, and $t_{l}^{w}=t_{2}^{w}-t_{1}^{w}$ is the length of the $w^{th}$ estimation window. Also, the overlap between the $w^{th}$ and ${\left(w+1\right)}^{th}$ estimation windows is defined as $t_{o}^{w,w+1}=t_{2}^{w}-t_{1}^{w+1},\;\forall \;w\leq n_{w}-1$. The estimation problem is solved at each estimation window iteratively to estimate the mean vector and covariance matrix of the unknown parameter vector and then moves to the next estimation window until completion. The estimates of the unknown model parameters at the end of each estimation window are transferred to the next estimation window and used as initial estimates. However, to transfer the estimates of FIMs, each estimation window is divided into two parts. The estimates of FIMs in the first part, spans from $t_{1}^{w}$ to $t_{1}^{w+1}$ in the $w^{th}$ estimation window, which does not overlap with the next estimation window, are considered as final estimates. The second part overlaps with the next estimation window, spans from $t_{1}^{w+1}$ to $t_{2}^{w}$ in the $w^{th}$ estimation window, and the estimates of FIMs in this part are transferred to the next estimation window to be considered as initial estimates. The sequential estimation window approach is schematically shown in Figure 2 and further discussed in the following section.

The FE-predicted response of a bridge at the $w^{th}$ estimation window, ${\hat{y}}_{t_{1}^{w}:t_{2}^{w}}={\left[\begin{array}{ccc} {\hat{y}}_{t_{1}^{w}}^{T} & {\hat{y}}_{t_{1}^{w}+1}^{T} & \begin{array}{cc} \ldots & {\hat{y}}_{t_{2}^{w}}^{T} \end{array} \end{array}\right]}^{T}\in {\mathbb{R}}^{(n_{y}\times t_{l}^{w})\times 1}$, to a general case of multiple-support earthquake excitation can be expressed as a nonlinear function of the model parameter vector, $\theta \in {\mathbb{R}}^{n_{\theta }\times 1}$, and the time history of the multiple-support FIMs, ${\ddot{u}}_{1:t_{2}^{w}}^{g}\in {\mathbb{R}}^{\left(t_{2}^{w}\times n_{s}\times 3\right)\times 1}$. This is shown in Equation (1). The terms $n_{\theta }$, $n_{s}$ and $n_{y}$ are, respectively, the number of model parameters, the number of supports with different translational input motions, and the number of measurement channels.

(1) $${\hat{y}}_{t_{1}^{w}:t_{2}^{w}}=h_{t_{1}^{w}:t_{2}^{w}}\left(\theta ,{\ddot{u}}_{1:t_{2}^{w}}^{g}\right)$$

In Equation (1), $h_{t_{1}^{w}:t_{2}^{w}}={\left[\begin{array}{ccc} h_{t_{1}^{w}}^{T} & h_{t_{1}^{w}+1}^{T} & \begin{array}{cc} \ldots & h_{t_{2}^{w}}^{T} \end{array} \end{array}\right]}^{T}$, $h_{t}\in {\mathbb{R}}^{n_{y}\times 1}$ is the nonlinear response function of the FE model at time step t, and ${\ddot{u}}_{1:t_{2}^{w}}^{g}={\left[\begin{array}{ccc} {\ddot{u}}_{1,1:t_{2}^{w}}^{g}{}^{T} & {\ddot{u}}_{2,1:t_{2}^{w}}^{g}{}^{T} & \begin{array}{cc} \ldots & {\ddot{u}}_{n_{s},1:t_{2}^{w}}^{g}{}^{T} \end{array} \end{array}\right]}^{T}$, in which ${\ddot{u}}_{n,1:t}^{g}\in {\mathbb{R}}^{\left(t\times 3\right)\times 1}$ is the time history of the three translational components of the FIMs at the $n^{th}$ support from time step 1 to time step t. The measured response vector of the bridge at the $w^{th}$ estimation window, $y_{t_{1}^{w}:t_{2}^{w}}\in {\mathbb{R}}^{(n_{y}\times t_{l}^{w})\times 1}$, is related to the FE-predicted response through a simulation error model as

(2) $$v_{t_{1}^{w}:t_{2}^{w}}\left(\theta ,{\ddot{u}}_{1:t_{2}^{w}}^{g}\right)=y_{t_{1}^{w}:t_{2}^{w}}-{\hat{y}}_{t_{1}^{w}:t_{2}^{w}}\left(\theta ,{\ddot{u}}_{1:t_{2}^{w}}^{g}\right)$$

in which $v_{t_{1}^{w}:t_{2}^{w}}\in {\mathbb{R}}^{(n_{y}\times t_{l}^{w})\times 1}$ is the simulation error vector at the $w^{th}$ estimation window and accounts for the misfit between the measured and FE-predicted responses of the bridge [23]. By neglecting the effects of modeling error, the simulation error at each time step t is ideally modeled as a spatially and temporally independent zero-mean Gaussian white noise process (i.e., $v_{t}\sim N\left(0,R\right)$). Hence, $v_{t_{1}^{w}:t_{2}^{w}}\sim N\left(0,{\tilde{R}}_{w}\right)$, where ${\tilde{R}}_{w}\in {\mathbb{R}}^{\left(t_{l}^{w}\times n_{y}\right)\times \left(t_{l}^{w}\times n_{y}\right)}$ is a block diagonal matrix whose block diagonals are the simulation error covariance matrix R. The objective of the model updating is to find the estimates of the unknown parameters for which the discrepancies between the measured and FE-predicted responses are minimized.

The unknown parameter vector at the $w^{th}$ estimation window is defined as $\varphi_{t_{1}^{w}:t_{2}^{w}}={\left[\theta^{T},{\ddot{u}}_{t_{1}^{w}:t_{2}^{w}}^{g}{}^{T}\right]}^{T}$, where $\varphi_{t_{1}^{w}:t_{2}^{w}}\in {\mathbb{R}}^{n_{\varphi ,w}\times 1}$ with $n_{\varphi ,w}=n_{\theta }+n_{s}\times t_{l}^{w}\times 3$. The unknown parameter vector is modeled as a random vector, the evolution of which is characterized by a Gaussian Markov process—also known as a random walk. A state-space model is set up, in which the state equation governs the evolution of the random unknown parameter vector and the measurement equation corresponds to the simulation error model [24], i.e.,

(3) $$\varphi_{t_{1}^{w}:t_{2}^{w},k+1}=\varphi_{t_{1}^{w}:t_{2}^{w},k}+\gamma_{w,k}$$

(4) $$y_{t_{1}^{w}:t_{2}^{w}}={\hat{y}}_{t_{1}^{w}:t_{2}^{w},k+1}\left(\varphi_{t_{1}^{w}:t_{2}^{w},k+1}\right)+v_{t_{1}^{w}:t_{2}^{w},k+1}$$

in which $\gamma_{w,k}\sim N\left(0,Q_{w}\right)$ is the process noise vector at the $k^{th}$ iteration of the $w^{th}$ estimation window, and $Q_{w}\in {\mathbb{R}}^{n_{\varphi ,w}\times n_{\varphi ,w}}$ is the process noise covariance matrix at the $w^{th}$ estimation window. Equations (3) and (4) represent a state-space model with unknown states (i.e., model parameters and FIMs herein). An Unscented Kalman Filtering (UKF) [25] method is used to estimate the unknown states. The estimation process is iterative at each estimation window. Therefore, the subscript k is added in Equations (3) and (4) to denote the iteration number.

To propagate the parameter uncertainties into the model, a scaled Unscented Transformation (UT) method is employed [26], in which the model is evaluated separately at a set of deterministically selected realizations of the parameter vector, which are referred to as the Sigma Points (SPs). The SPs at the $w^{th}$ estimation window are selected based on the prior mean vector (${\hat{\varphi }}_{t_{1}^{w}:t_{2}^{w}}^{-}$) and prior covariance matrix (${\left({\hat{P}}_{\varphi \varphi }^{-}\right)}_{t_{1}^{w}:t_{2}^{w}}$) of the unknown parameters. The vector of SPs at the $w^{th}$ estimation window is defined as $\vartheta_{w}={\left[\begin{array}{ccc} \vartheta_{w}^{1} & \vartheta_{w}^{2} & \begin{array}{cc} \ldots & \vartheta_{w}^{2n_{\varphi ,w}+1} \end{array} \end{array}\right]}^{T}$. The mean vector (${\bar{y}}_{t_{1}^{w}:t_{2}^{w}})\;$and covariance matrix of the FE-predicted responses (${\left({\hat{P}}_{yy}\right)}_{t_{1}^{w}:t_{2}^{w}})$ at the $w^{th}$ estimation window, as well as the cross-covariance matrix of vectors $\varphi_{t_{1}^{w}:t_{2}^{w}}$ and $y_{t_{1}^{w}:t_{2}^{w}}$, shown as ${\left({\hat{P}}_{\varphi y}\right)}_{t_{1}^{w}:t_{2}^{w}}$, are computed using a weighted sampling method as follows.

(5) $${\bar{y}}_{t_{1}^{w}:t_{2}^{w}}=\overset{2n_{\varphi ,w}+1}{\underset{j=1}{\sum }}W_{m}^{j}{\hat{y}}_{t_{1}^{w}:t_{2}^{w}}\left(\vartheta_{w}^{j}\right)$$

(6) $${\left({\hat{P}}_{yy}\right)}_{t_{1}^{w}:t_{2}^{w}}=\overset{2n_{\varphi ,w}+1}{\underset{j=1}{\sum }}W_{e}^{j}\left[{\hat{y}}_{t_{1}^{w}:t_{2}^{w}}\left(\vartheta_{w}^{j}\right)-{\bar{y}}_{t_{1}^{w}:t_{2}^{w}}\right]{\left[{\hat{y}}_{t_{1}^{w}:t_{2}^{w}}\left(\vartheta_{w}^{j}\right)-{\bar{y}}_{t_{1}^{w}:t_{2}^{w}}\right]}^{T}+{\tilde{R}}_{w}$$

(7) $${\left({\hat{P}}_{\varphi y}\right)}_{t_{1}^{w}:t_{2}^{w}}=\overset{2n_{\varphi ,w}+1}{\underset{j=1}{\sum }}W_{e}^{j}\left[\vartheta_{w}^{j}-{\hat{\varphi }}_{t_{1}^{w}:t_{2}^{w}}^{-}\right]{\left[{\hat{y}}_{t_{1}^{w}:t_{2}^{w}}\left(\vartheta_{w}^{j}\right)-{\bar{y}}_{t_{1}^{w}:t_{2}^{w}}\right]}^{T}$$

**Figure 3:**
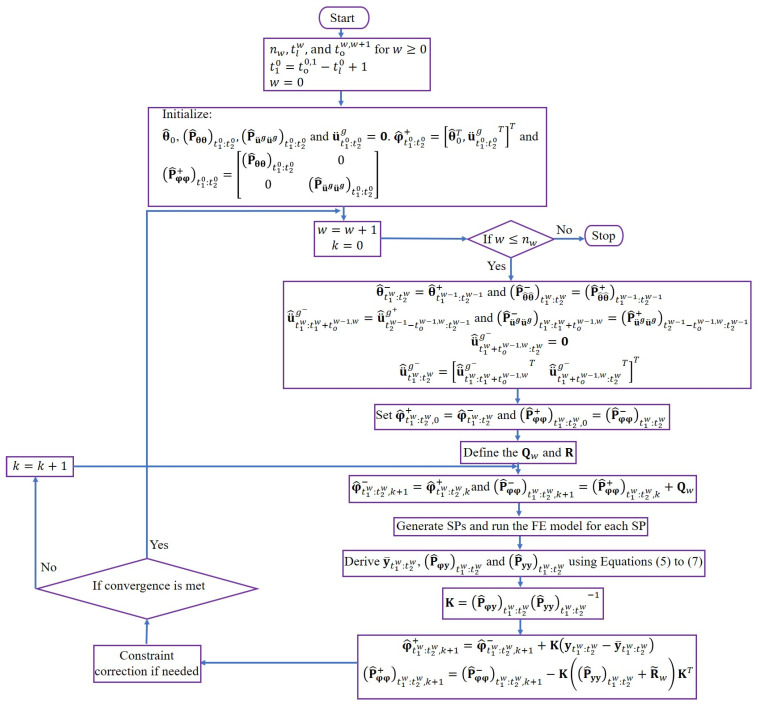
Sequential Bayesian FE model updating flowchart based on the UT method for joint estimation of the unknown model parameters and the FIM time histories.

In Equations (5)–(7), $W_{m}^{j}$ and $W_{e}^{j}$ are the mean and covariance weighting coefficients [26], respectively, and ${\hat{y}}_{t_{1}^{w}:t_{2}^{w}}\left(\vartheta_{w}^{j}\right)$ is the FE-predicted response at the $w^{th}$ estimation window evaluated at $\vartheta_{w}^{j}$. Now, the UKF prediction-correction procedure can be employed to estimate the posterior mean vector ${\hat{\varphi }}^{+}{}_{t_{1}^{w}:t_{2}^{w},k+1}$ and posterior covariance matrix ${\left({\hat{P}}_{\varphi \varphi }^{+}\right)}_{t_{1}^{w}:t_{2}^{w},k+1}$ of parameter vector at the ${\left(k+1\right)}^{th}$ iteration. Moreover, to move from the $w^{th}$ estimation window to the ${\left(w+1\right)}^{th}$ estimation window, convergence criteria, including the maximum number of iterations at each estimation window and allowable convergence tolerance in the posterior mean vector, are checked. To avoid unphysical estimates of posterior parameters, a constrain correction approach based on [27] is also implemented. The model updating algorithm is summarized in Figure 3.

### 2.2. Formulation for the Identifiability Analysis

Successful estimation of the unknown model parameters depends on the information that the measurement data may contain about those model parameters, as well as the sensitivity of the FE model responses with respect to those parameters. To quantify the information content of the measurement data and, therefore, to assess the identifiability of the model parameters, an approach similar to one presented in [28] is used. In this approach, the information gain of the $i^{th}$ model parameter $\left(\theta^{i}\right)$ from measurement data, $\Delta H\left(\theta^{i}\right)$**,** is expressed as the difference between the *a priori* and *a posteriori* information entropy, which can be calculated as

(8) $$\Delta H\left(\theta^{i}\right)=\frac{1}{2}\ln\left({\left[I\right]}_{ii}p_{i}+1\right)$$

where $p_{i}$ is the *a priori* variance of $\theta^{i}$ and ${\left[I\right]}_{ii}$ is the $i^{th}$ diagonal element of the Fisher Information Matrix defined as

(9) $$I=\overset{n}{\underset{t=1}{\sum }}{\left.{\left(\frac{\partial {\hat{y}}_{t}}{\partial \theta }\right)}^{T}\right|}_{\theta =\hat{\theta }}R^{-1}{\left.\frac{\partial {\hat{y}}_{t}}{\partial \theta }\right|}_{\theta =\hat{\theta }}$$

in which n is the total number of time steps. The term **R** is the covariance matrix of the simulation error vector as defined earlier and $\hat{\theta }$ is *a maximum a posteriori* (MAP) estimate, which is approximated with the initial estimates based on the recommendations provided in [28]. To calculate the sensitivity terms (i.e., $\partial {\hat{y}}_{t}/\partial \theta$), a Finite Difference Method is employed. Then, the information gain of different model parameters is compared to sort out the model parameters with the highest information gain, which are relatively more likely to be identifiable unless they have strong dependence on other model parameters. The mutual entropy gain between $\theta^{i}$ and $\theta^{j}$, $\Delta M\left(\theta^{i},\theta^{j}\right)$**,** can be quantified through a mutual gain metric defined as

(10) $$\Delta M\left(\theta^{i},\theta^{j}\right)=\frac{1}{2}\ln\left[\frac{\left({\left[I\right]}_{ii}+p_{i}{}^{-1}\right)\left({\left[I\right]}_{jj}+p_{j}{}^{-1}\right)}{\left|{\left[I\right]}_{ij}+P_{ij}{}^{-1}\right|}\right],\;\mathrm{with}\;\tilde{I}=\left[\begin{array}{cc} {\left[I\right]}_{ii} & {\left[I\right]}_{ij}\\ {\left[I\right]}_{ji} & {\left[I\right]}_{jj} \end{array}\right]$$

where $P_{ij}$ is the *a priori* covariance matrix of $\theta^{i}$ and $\theta^{j}$. In case of a strong dependency between two parameters, the model parameter with smaller information gain (based on Equation (8)) is fixed and the other model parameter will be estimated.

## 3. Verification Case Study Using the San Roque Canyon Bridge

**Figure 4:**
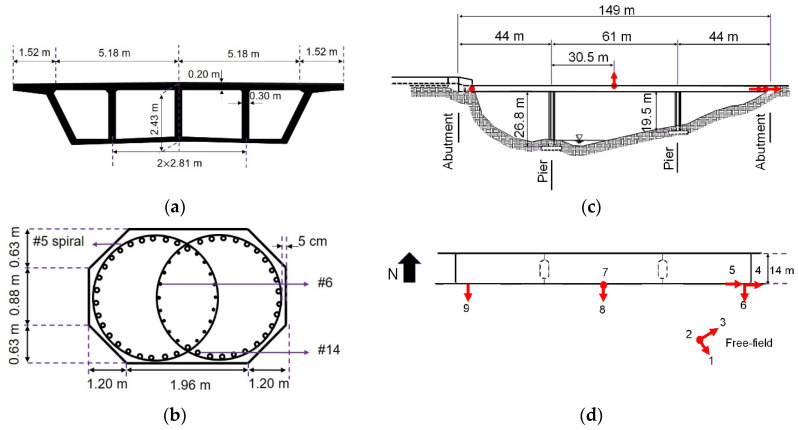
SRC bridge and its instrumentation layout [29]: (**a**) deck cross-section, (**b**) pier cross-section, (**c**) elevation view, and (**d**) plan view and installed accelerometers.

A precast reinforced concrete bridge, referred to as the San Roque Canyon (SRC) bridge, is used as a testbed to examine the efficacy of the model updating technique. The SRC bridge, located in Santa Barbara County, CA, crosses the San Roque Creek river and is a 149-m long continuous concrete box girder bridge with a 14-m wide deck and two lanes of traffic. The SRC bridge has two concrete piers with octagonal cross-section and seat-type abutments. The bridge deck and pier cross-sections are shown in Figure 4a,b. This bridge was instrumented in 1996 with six uniaxial accelerometers on the bridge and three uniaxial accelerometers at a nearby free-field station, see Figure 4c,d. Three out of six accelerometers on the bridge measure the response of the deck in the transverse direction (channels 6, 8, and 9), and one accelerometer records the vertical motion of the deck at its center (channel 7). The channels 4 and 5 record the longitudinal response of the abutment and deck, respectively. Also, channels 1, 2, and 3 are at the free-field to collect FFMs in the horizontal (channels 1 and 3) and vertical directions (channel 2).

**Figure 5:**
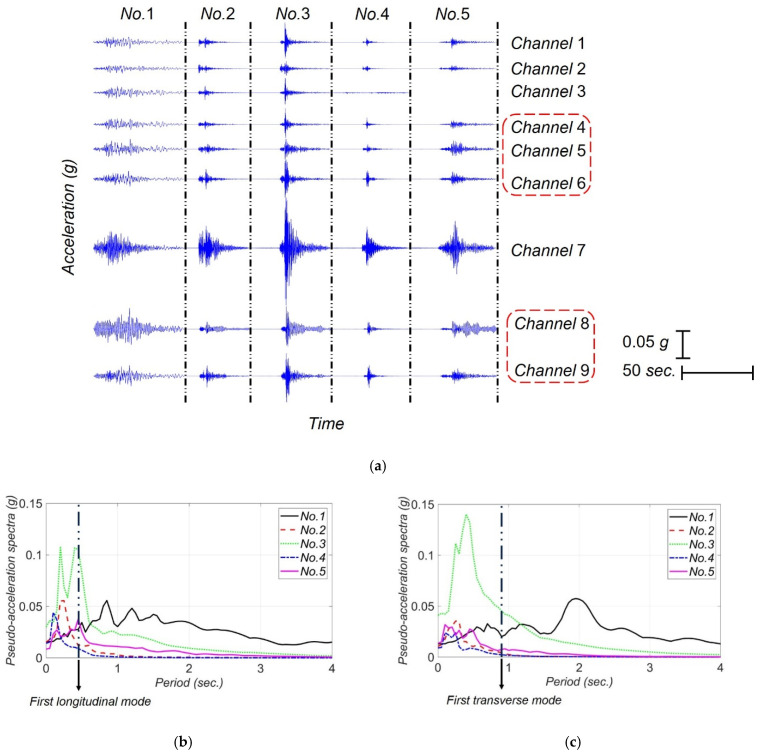
Earthquake data used for model updating: (**a**) recorded acceleation responses at the SRC bridge for earthquakes listed in Table 1, (**b**) pseudo spectral acceleration of FIMs projected in the longitudinal direction, and (**c**) pseudo spectral acceleration of FIMs projected in the transverse direction. The period of the first longitudinal and transverse modes of the SRC (calculated based on the prior model) are shown in the figure.

Since its instrumentation until 2021, SRC bridge has recorded seven earthquakes whose data is publicly available through the Center for Engineering Strong Motion Data (CESMD) [30]. However, only five earthquakes with Peak Ground Acceleration (PGA) greater than 0.01 g are available and all five earthquakes are considered in this study. These five earthquakes are listed in Table 1 along with their date, distance between the epicenter and the bridge, PGA, and Peak Structural Accelerations (PSAs) in different directions. The PGA for all the earthquakes, except for the 2004 IslaVista, are in horizontal direction. The recorded acceleration data for these five earthquakes are shown in Figure 5a, in which the measurement channels used later for the identifiability analysis and model updating are specified by red dashed lines. Moreover, the pseudo spectral acceleration for FFMs in transverse and longitudinal directions considering 5% damping ratio are shown in Figure 5b,c. The period of the first longitudinal and transverse modes of SRC are also shown in this figure.

### 3.1. FE Model of the SRC Bridge

**Figure 6:**
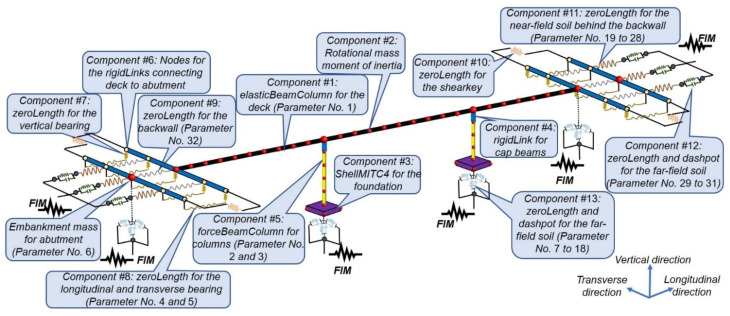
Schematic representation of the SRC bridge FE model. The parameter numbers in the parentheses are listed in Table 2.

A detailed FE model of the SRC bridge is developed in OpenSees [31] following the guidelines provided in [32]. All the nominal material properties are taken from the as-built structural drawings and Caltrans Seismic Design Criteria [33]. Figure 6 is a schematic representation of the FE model, in which the model components are numbered from 1 to 13 and explained in the following text. Moreover, the model parameters are numbered from 1 to 34 and their nominal values are listed in Table 2.

The deck (Component #1) is modeled using *elasticBeamColumn* elements, as it is a capacity protected element [33]. Each span is meshed using ten elements; the cross-sectional properties are calculated based on the section geometry shown in Figure 4a and the material modulus of elasticity ($E_{d}$) is 27.8 GPa. To account for the torsional vibrations, the rotational mass moment of inertia is calculated based on the section geometry and is added to deck nodes (Component #2). The pier foundations are modeled using four 4 m × 4 m linear-elastic *shellMITC4* elements (Component #3) with 1.7 m thick *ElasticMembranePlateSection* section and modulus of elasticity of 27.8 GPa. To connect columns to the deck, *rigidLink* elements (Component #4) are used to account for the rigidity provided by the cap beams. Fiber-section *forceBeamColumn* elements with five integration points [34] are utilized to model the piers (Component #5). The elastic shear and torsional stiffnesses are aggregated to the pier fiber sections. The confinement effects in the pier sections are considered using the Mander’s model [35]. The confined core is modeled using *Concrete04* with compressive strength $\left(f_{c,c}^{’}\right)$ of 40.4 MPa achieved at 0.37% strain and initial modulus of elasticity ($E_{c}$) of 27.8 GPa. Moreover, reinforcement is modeled using *Steel02* material with a yield strength of 46.9 GPa and a modulus of elasticity of 200 GPa. To connect the deck to the abutment system, rigid frame elements (Component #6) are added to the two ends of the deck with the number of nodes equal to the number of bearing pads. These nodes are connected to the rigid abutment body through bearing pads and shear keys. Bearing pads in the vertical direction are modeled using *zeroLength* elements with *ElasticPPGap* uniaxial material with compression-only vertical compliance up to a 0.01 m deformation, known as the initial gap (Component #7). The stiffness in bearing pads (Component #8) is modeled using *zeroLength* elements with uniaxial bilinear *steel01* material assuming$\;10^{8}\;\frac{N}{m}$ and $10^{7}$ $\frac{N}{m}$ shear stiffness in the longitudinal $\left(k_{L}^{b}\right)$ and transverse $\left(k_{T}^{b}\right)$ directions, respectively, and 1% strain-hardening ratio. Once the gap between the deck and the abutment backwall is closed, the backwall provides resistant in the longitudinal direction. The backwall stiffness (Component #9), which is calculated using the theory of plate and shell [36], is modeled using compression-only elastic perfectly-plastic gap behavior. For this purpose, an *ElasticPPGap* uniaxial material with a 0.05 m gap is used. The transverse response of the deck is controlled by the *zeroLength* elements representing the shear keys (Component #10) with a shear stiffness of 100 MN/m. According to the structural drawings, the shear keys of SRC bridge are ductile. So, the model proposed in [37] can be used to model the nonlinear compression-only behavior of the shear keys after the gap between the deck and shear keys is closed. However, since the level of excitation in this study is low, the shear keys are not modeled and it is assumed that their stiffness is lumped to the stiffness of bearing pads.

SSI effects are modeled to consider near-field and far-field effects [19,20,38]. At the abutments, the near-filed effects include the passive soil pressure behind the backwall, which provides resistance against the abutment movement. This passive soil pressure is modeled using *zeroLength* soil springs (Component #11) with a *HyperbolicGapMaterial* based on the Generalized Hyperbolic Force–Displacement (GHFD) backbone curve [39]. The backbone curve is developed using the abutment wall height (2.44 m), wall-soil interface friction angle ($40^{{}^{\circ}}$), and Mohr-Coulomb shear strength parameters with a common silty-sand soil type [40], whose properties are taken from [41]. The calculated values for the stiffness in the vertical $\left(k_{V}^{a}\right)$, longitudinal $\left(k_{L}^{a}\right)\;$and transverse $\left(k_{T}^{a}\right)$ directions are equal to 10, 8.7 and 37 GN/m, respectively. The vertical $\left(c_{V}^{a}\right)$, longitudinal $\left(c_{L}^{a}\right)$, and transverse $\left(c_{T}^{a}\right)$ damping coefficients of soil dashpots are set to 150, 170, and 130 MN.s/m, respectively. The rotational stiffness about the longitudinal $\left(k_{R,L}^{a}\right)$ and vertical $\left(k_{R,V}^{a}\right)$ axis is 300 and 240 GN.m/rad. In addition, the rotational damping coefficients about the vertical $\left(c_{R,V}^{a}\right)$ and longitudinal $\left(c_{R,L}^{a}\right)$ axis are 390 and 4.7 GN.m.s/rad, respectively. The far-field soil-embankment effect is modeled through three parallel *zeroLength* spring and dashpot elements (Component #12), with the stiffness and damping properties calculated based on [42]. The springs have elastic-no-tension (*ENT*) uniaxial material with a modulus of elasticity $\left(k_{L}^{fs}\right)$ equal to 3 GPa. The dashpots have *Viscous* uniaxial material with a damping coefficient defined as the summation of linear radiation $\left(c_{R}^{fs}\right)$ and material damping $\left(c_{L}^{fs,m}\right)$ coefficients. The parameters $c_{R}^{fs}$ and $c_{L}^{fs,m}$ are taken as 38 and 140 MN.s/m, respectively.

At the foundations, the near-field SSI effects are neglected and the far-field effects under the piers and abutments foundations (Component #13) in all six degrees of freedom are modeled using *zeroLength* elements (springs and dashpots) with stiffness and damping coefficients calculated based on [43]. Corresponding vertical $\left(k_{V}^{p}\right)$, longitudinal $\left(k_{L}^{p}\right)$, and transverse $\left(k_{T}^{p}\right)$ stiffnesses are set to 12.7, 9.8, and 9.8 GN/m, respectively. Also, the vertical $\left(c_{V}^{p}\right)$, longitudinal $\left(c_{L}^{p}\right)$, and transverse $\left(c_{T}^{p}\right)$ damping coefficients are set to 240, 220, and 190 MN.s/m, respectively. In addition, the rotational stiffness about the vertical $\left(k_{R,V}^{p}\right)$, longitudinal $\left(k_{R,L}^{p}\right)$, and transverse $\left(k_{R,T}^{p}\right)$ axes are equal to 290, 170, and 170 GN.m/rad, respectively. Similarly, the rotational damping coefficients about the vertical $\left(c_{R,V}^{p}\right)$, longitudinal $\left(c_{R,L}^{p}\right)$, and transverse $\left(c_{R,T}^{p}\right)$ axes are equal to 3.6, 2.7, and 2.7 GN.m.s/rad, respectively.

In addition to the explicit sources of damping energy dissipation in the model (dashpots and nonlinear materials), the inherent damping property of the structure is modeled using Rayleigh damping with the mass ($a_{0}$) and stiffness ($a_{1}$) proportional coefficients equal to 0.6 and 0.003, respectively, which results in 5% damping for modes 1 and 6. The Rayleigh damping is modeled using the initial stiffness matrix.

### 3.2. Identifiability Analysis

An identifiability analysis is performed to find the most identifiable model parameters based on the approach described in Section 2.2. The identifiability analysis is an input-output approach measuring the sensitivity of model responses with respect to different model parameters. The analysis is completed here using the significant portion of the 2004 Isla Vista earthquake, and the FFMs are used in lieu of FIMs. Based on the instrumentation plan in Figure 4, channel 7 records the vertical component of the bridge response and is likely polluted by the traffic-induced vibrations and thus not included for the identifiability analysis. Consequently, the vertical component of the input motion (channel 2), is also removed from the analysis. The identifiability analysis process is discussed in the following part.

**Figure 7:**
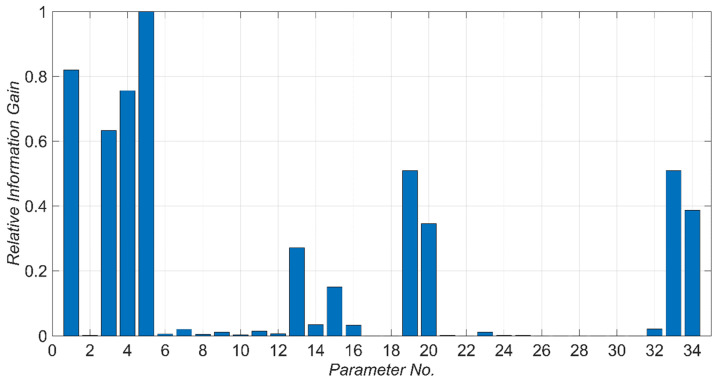
Relative information gain of model parameters. Parameters are defined in Table 2.

**Figure 8:**
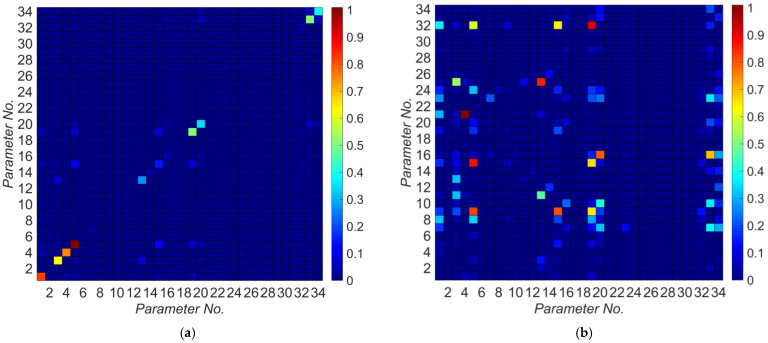
Relative mutual entropy gain between model parameter pairs: (**a**) without scaling, (**b**) with scaling.

A list of potential model parameters and their nominal values are shown in Table 2. The longitudinal, transverse, and vertical directions mentioned in this table are based on the directions shown in Figure 6. Model parameters are numbered from 1 to 34. These parameter numbers are used in Figure 6 to relate the model parameters to the corresponding model components. To find the most identifiable model parameters, the relative information gain for each model parameter is calculated based on Equation (8) and the results are shown in Figure 7. As can be seen in this figure, there are 10 model parameters that are potentially identifiable due to their high relative information gain in comparison to other model parameters. These model parameters, sorted in descending order with respect to their relative information gain, are: $k_{L}^{b}$, $E_{d}$, $k_{T}^{b}$, $E_{c}$, $k_{L}^{a}$, $a_{0}$, $a_{1}$, $c_{L}^{a}$, $k_{R,L}^{p}$, and $k_{R,T}^{p}$. As can be seen in Figure 7, the model parameters related to the nonlinear response of bridge, including $f_{c,c}^{’}$, are likely unidentifiable. This is because the considered earthquake in this study has small amplitude. Figure 8a shows the relative mutual information gain among the model parameter pairs obtained from Equation (10). Moreover, to better observe the dependency between model parameters, a scaled version of Figure 8a is presented in Figure 8b. In this figure, all the mutual information gains in each row are scaled to the maximum value in the corresponding row. Also, the diagonal values are nullified (actual values are replaced by zero). As can be seen, there is no significant dependence between the first four model parameters with high relative information gain ($k_{L}^{b}$, $E_{d}$, $k_{T}^{b}$, and$\;E_{c}$). So, these parameters are chosen as unknown model parameters to be estimated through the model updating process. Parameters $k_{R,T}^{p}$ and $k_{R,L}^{p}$ are removed from the unknowns because they are dependent on parameters $k_{L}^{b}$ and $E_{c}$, respectively. Furthermore, parameters $a_{0}$ and $a_{1}$ are moderately dependent; however, both are kept among the unknowns to be estimated. Parameters $k_{L}^{a}$ and $c_{L}^{a}$ are dependent on $E_{d}$ and $k_{L}^{b}$. This is likely due to the competing effects that parameters $k_{L}^{a},\;E_{d}$, and $k_{L}^{b}$ have on the stiffness of the superstructure in the longitudinal direction. Since $E_{d}$ is already selected as an unknown model parameter to be estimated, $k_{L}^{a}$ and $c_{L}^{a}$ are excluded from the unknown parameter vector for the verification studies. In summary, parameters $E_{d}$, $E_{c}$, $k_{T}^{b}$, $k_{L}^{b}$, $a_{0}$, and $a_{1}$, highlighted in Table 2, are selected as unknown model parameters to be estimated.

### 3.3. Verification Study Using Numerically Simulated Data

To simulate the measurements, the FE response of the SRC bridge to the 2004 Isla Vista earthquake is simulated and polluted with an artificial zero-mean Gaussian noise with 5% Root Mean Square (RMS) noise-to-signal ratio. For the simulation, the measured free field motions are used as the FIMs. The time history analysis is performed using time step size of 0.01 sec and the Newmark-beta average acceleration method for time integration, with the nominal model parameter values presented in Table 2. The noisy simulated responses are used for model updating to estimate the six unknown model parameters and the FIM time histories in the longitudinal and transverse directions. It should be noted that due to the short length of the SRC bridge, the FIMs are assumed to be uniform. As discussed before, measurement channels 7 and 2 are not considered and only channels 4, 5, 6, 8, and 9 are used in the model updating.

The model updating is carried out using 31 number of overlapping estimation windows ($n_{w}=31$). The length of each estimation window is 27 time steps ($t_{l}^{w}=27$), and the overlap between each two estimation windows is equal to 10 time steps ($t_{o}^{w,w+1}=10$). In order to initialize the model parameter vector (${\hat{\theta }}_{0}$), −20% estimation error is assumed in the initial estimates of all unknown model parameters where estimation error is defined as

(11) $$\mathrm{Estimation}\;\mathrm{error}\left(\%\right)=\left(\frac{\mathrm{Estimated}\;\mathrm{model}\;\mathrm{parameter}\;\mathrm{value}}{\mathrm{Nominal}\;\mathrm{model}\;\mathrm{parameter}\;\mathrm{value}}-1\right)\times 100$$

and the nominal values are shown in Table 2. The assumed −20% deviation of model parameters from their nominal values is chosen as a reasonable initial error based on engineering judgement. The final estimates of the model parameters are sensitive to the initial estimates. However, as long as the initial estimates are not unreasonably far from the nominal values, no significant change in the final estimates is expected [44]. The term ${\left({\hat{P}}_{\theta \theta }\right)}_{t_{1}^{0}:t_{2}^{0}}$ is set as a diagonal matrix with $i^{th}$ diagonal entry equal to ${\left(0.1{\hat{\theta }}_{0,i}\right)}^{2}$, while ${\hat{\theta }}_{0,i}$ is the $i^{th}$ entry of ${\hat{\theta }}_{0}$. Moreover, ${\left({\hat{P}}_{{\ddot{u}}^{g}{\ddot{u}}^{g}}\right)}_{t_{1}^{0}:t_{2}^{0}}$ is also initialized as a $t_{l}^{0}\times t_{l}^{0}$ diagonal matrix with diagonal entries equal to ${\left(0.001g\right)}^{2}$. The first $n_{\theta }$ diagonal entries in $Q_{w}$ are equal to ${\left(0.001{\hat{\theta }}_{w,i}\right)}^{2}$, where ${\hat{\theta }}_{w,i}$ is the $i^{th}$ entry of the$\;{\hat{\theta }}_{w}$, and the rest are time-invariant and equal to $10^{-8}$—note that the term ${\hat{\theta }}_{w}$ refers to the estimate of model parameter vector at the $w^{th}$ estimation window. Finally, the diagonal entries of the simulation error covariance matrix, R*,* representing the measurement noise variances, are set as ${\left(0.003\%g\right)}^{2}$. Moreover, the maximum number of iterations and the allowable convergence tolerance are selected equal to 9 and 0.02. These thresholds are based on experience to achieve a compromise between estimation accuracy and computational demand.

**Figure 9:**
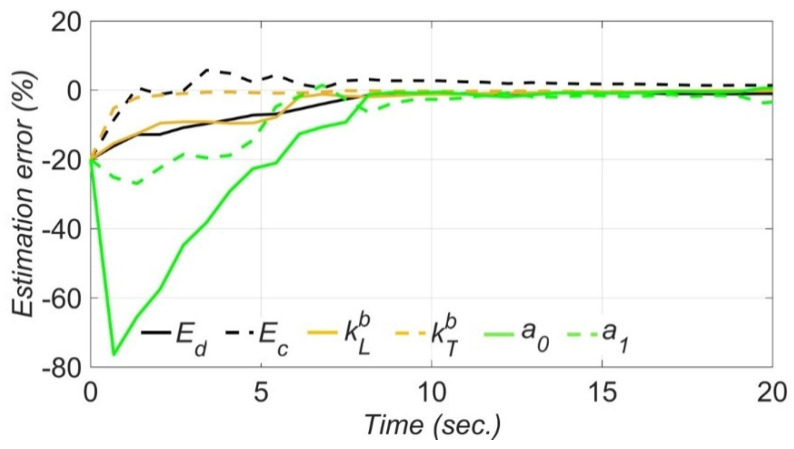
Estimation error time histories for the unknown model parameters through the model updating technique using numerically simulated data.

The time histories of the estimation error for unknown model parameters are shown in Figure 9. In this figure, the error in the estimation of model parameters are calculated at the end of each estimation window. As can be seen, the estimation errors for most unknown model parameters are significant in the first five seconds. However, as time passes and more information is collected, the estimation errors decrease and converge to zero with less than 4% error. Also, as seen in this figure, elastic modulus of deck and initial elastic modulus of columns converge to their nominal values faster than the other unknown model parameters. This is because they are associated with the initial linear-elastic stiffness of the bridge structural components.

**Figure 10:**
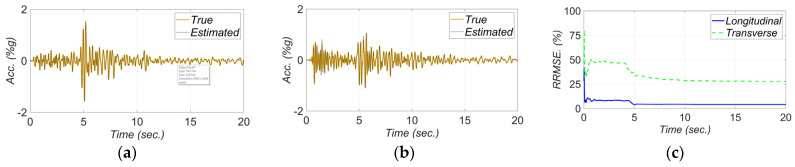
Comparison between true and estimated FIMs in (**a**) longitudinal and (**b**) transverse directions, and (**c**) RRMSE of the estimated FIMs.

The estimated FIMs in the longitudinal and transverse directions are compared to their true counterparts in Figure 10a,b, respectively. Moreover, to evaluate the accuracy of the estimated FIMs, the Relative Root Mean Square Error (RRMSE) of the estimated FIM time histories are shown in Figure 10c. The RRMSE at time $t_{n}$ is calculated as

(12) $$\mathrm{RRMSE}.\;\left(\%\right)=\frac{\sqrt{\sum_{i=t_{1}}^{t_{n}}{\left({\hat{s}}_{i}-s_{i}\right)}^{2}}}{\sqrt{\sum_{i=t_{1}}^{t_{n}}{\left(s_{i}\right)}^{2}}}\times 100$$

where the terms ${\hat{s}}_{i}$ and $s_{i}$ are the estimated and true time history values at the $i^{th}$ time step, respectively. As seen in Figure 10, the estimated FIMs are less accurate at the beginning of estimation, when model parameters are not accurately estimated yet, and as time passes the RRMSEs decrease.

**Figure 11:**
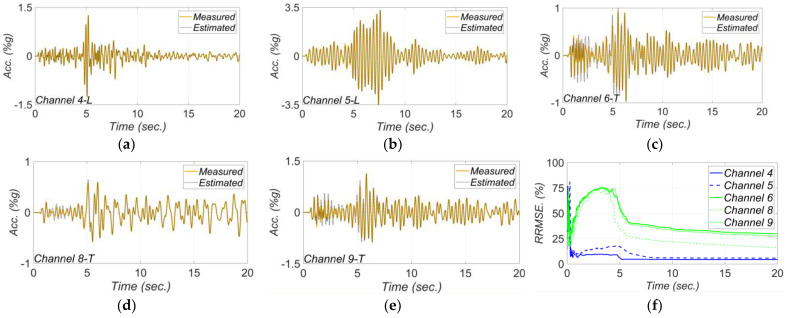
Comparison between measured and estimated responses obtained from the updated FE model at: (**a**) channel 4, (**b**) channel 5, (**c**) channel 6, (**d**) channel 8, (**e**) channel 9. Part (**f**) is the corresponding RRMSEs.

Figure 11 shows a comparison between measured and estimated responses at the measurement channels. As can be seen, the match between the estimated and measured response time histories is reasonable, which verifies the model updating technique. The RRMSE values for the estimated responses decrease in time due to improved accuracy in the estimated FIMs and unknown model parameters.

## 4. Case Study Using Real-World Earthquake Data

In this section, the SRC bridge model is integrated with the real data collected during earthquakes shown in Table 1. Using the presented model updating technique, the 6 unknown model parameters, already specified in Section 3.2, are estimated jointly with the two FIMs in the longitudinal and transverse directions. Details of the model updating process is similar to those explained in the previous section, and the nominal parameter values (shown in Table 2) are used as the initial estimates for the unknown model parameters.

**Figure 12:**
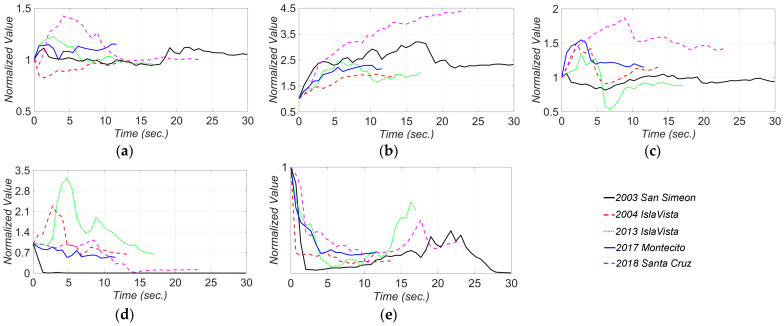
Estimation time histories of (**a**) $E_{ave}$, (**b**) $k_{L}^{b}$, (**c**) $k_{T}^{b}$, (**d**) $a_{0}$, and (**e**) $a_{1}$ for different earthquakes. The estimates are normalized to the initial estimates.

**Figure 13:**
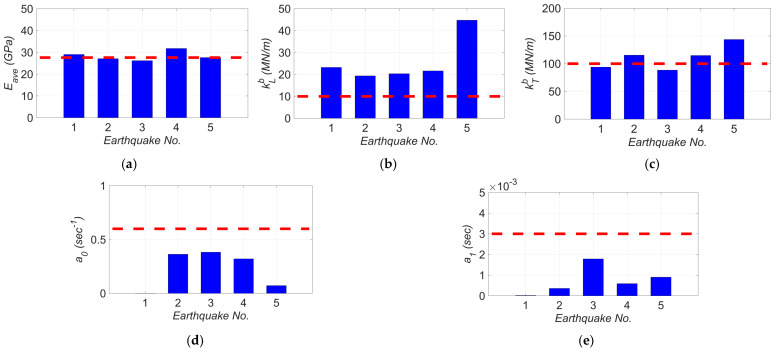
The variation in final estimates of (**a**) $E_{ave}$, (**b**)$\;k_{L}^{b}$, (**c**)$\;k_{T}^{b}$, (**d**) $a_{0}$, and (**e**) $a_{1}$ for different earthquakes. The red dashed lines show the nominal values.

Figure 12 shows the estimation time history of the unknown model parameters. In this figure, the parameters’ estimates are normalized to their nominal (initial) values, and the term $E_{ave}$ is the average of estimates for the concrete modulus of elasticity in deck ($E_{d})\;$and the initial modulus of elasticity in columns $\left(E_{c}\right)$. The event-to-event variabilities in the final estimates of the unknown model parameters are shown in Figure 13. In this figure, the nominal values are shown as dashed red lines. As seen in Figure 13a, while the final estimates for the concrete modulus of elasticity are close to the nominal values, there is minor event-to-event variability. These minor variations could be due to the concrete aging, modeling uncertainties, and other sources of modeling error. The variation in the final estimates of elastomeric bearing pad’s shear stiffness in longitudinal and transverse directions are shown in Figure 13b and Figure 13c, respectively. As can be seen, the level of the final estimates in the transverse direction is higher than the ones in the longitudinal direction. This is likely because the model parameter $k_{T}^{b}$ represents the combined effects of the stiffness of the elastomeric pad and shear key, while the model parameter $k_{L}^{b}$ only accounts for stiffness of the elastomeric pad. The level of the final estimates in Figure 13b,c are close to each other for different earthquakes with the exception of the 2018 Santa Cruz earthquake (earthquake No. 5). This variation can be due to the fact that the 2018 Santa Cruz earthquake is one of the weakest events used in this study with small PGA and PSAs (see Table 1). In such a weak event, the bearing pad stiffness is likely high enough to prevent any movement. In this case, the measurements may not be sensitive to the stiffness of the bearing pads, which could result in large estimates for $k_{L}^{b}$ and $k_{T}^{b}$.

**Figure 14:**
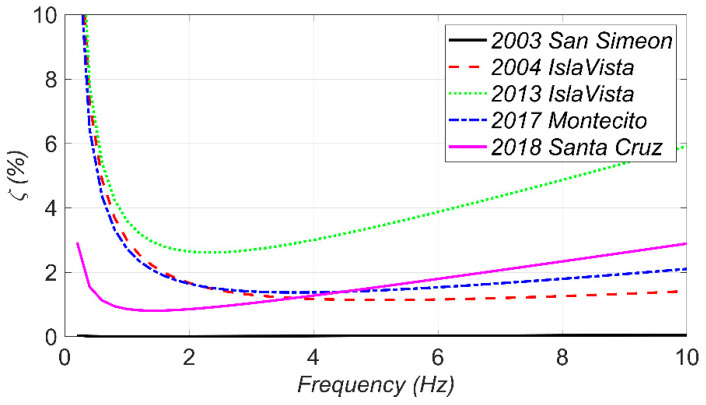
The final estimates of the Rayleigh damping ratio as a function of freqeuncy for different earthquakes.

To better investigate the estimated Rayleigh damping, the final estimates of the damping coefficients ($a_{0}$ and $a_{1}$) are used to calculate the damping ratio as a function of frequency as shown in Figure 14. As can be seen, the model updating results show almost no damping during the first earthquake, i.e., 2003 San Simeon. This is because that the bridge behaves almost quasi-statically in this excitation due to the superior presence of low-frequency components in 2003 San Simeon in comparison to the other earthquakes (see Figure 5). On the other hand, the highest damping ratios are estimated from the 2013 Isla Vista earthquake with the highest PGA, which suggests a correlation between the inherent damping and earthquake intensity. Based on these results, considering a 3–4% Rayleigh damping ratio for the first and eighth modes (with frequencies of 1.12 and 5.82 Hz, respectively) can be a rational assumption for the SRC bridge in the case of low to moderate earthquakes, i.e., 2013 Isla Vista. However, under weak excitations, e.g., the 2004 Isla Vista earthquake, lower damping ratio, around 1–2%, can be considered for these modes.

**Figure 15:**
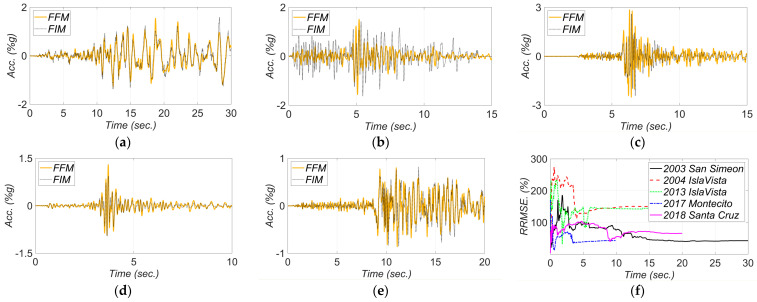
Comparison between the recorded FFMs and estimated FIMs in the longitudinal direction for (**a**) 2003 San Simeon, (**b**) 2004 IslaVista, (**c**) 2013 IslaVista, (**d**) 2017 Montecito, and (**e**) 2018 Santa Cruz, earthquake events. Part (**f**) is the RRMSE between the estimated FIMs and recorded FFMs.

**Figure 16:**
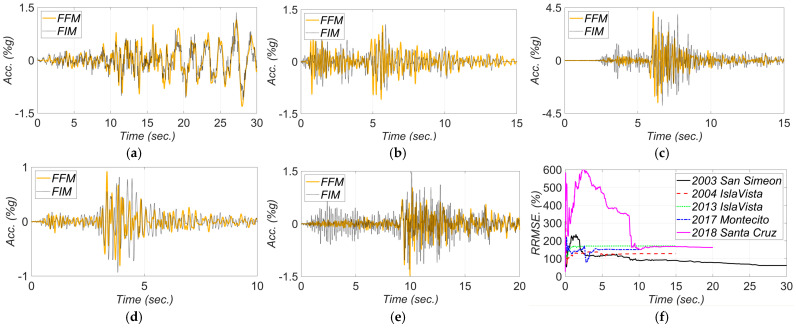
Comparison between the recorded FFMs and estimated FIMs in the transverse direction for (**a**) 2003 San Simeon, (**b**) 2004 IslaVista, (**c**) 2013 IslaVista, (**d**) 2017 Montecito, and (**e**) 2018 Santa Cruz, earthquake events. Part (**f**) is the RRMSE between the estimated FIMs and recorded FFMs.

The comparisons between the FFMs and the estimated FIMs and the corresponding RRMSEs in the longitudinal and transverse directions are shown in Figure 15 and Figure 16, respectively. Here, RRMSE presents the misfit between the predicted FIMs and collected FFMs. So, greater values for RRMSEs can imply more significant SSI effects. Note that as channel 3 malfunctioned during the 2017 Montecito and 2018 Santa Cruz earthquakes, measurements of channels 4 and 1 are considered to report the recorded FFMs in the longitudinal and transverse directions, respectively, for these two earthquakes. In Figure 15, the estimated FIMs in the longitudinal direction are compared with the recorded measurements in channel 4, and as can be seen, these two signals match well for these two earthquakes. In other words, what is recorded by measurement channel 4 on the abutment overlays the estimated FIM. This means that there is likely no significant bridge-abutment interaction. The best fit between the FIMs and FFMs is seen for the 2003 San Simeon earthquake, which is not surprising due to the low-frequency content of this motion as discussed earlier. Overall, the estimated longitudinal FIMs in all earthquakes show close similarity with the recorded FFMs. Therefore, neither kinematic interaction nor inertial interaction is likely significant for this bridge under the studied ground motion intensities in the longitudinal direction; however, a higher level of differences is observed between recorded FFMs and estimated FIMs in the transverse direction.

**Figure 17:**
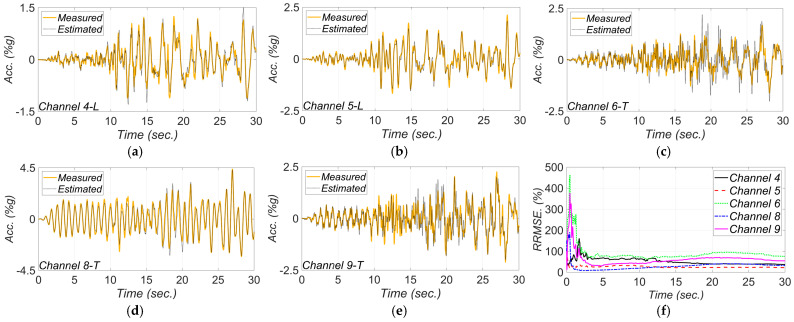
Comparison between measured and estimated (from updated model) responses at different measurement channels for 2003 San Simeon earthquake: (**a**) channel 4, (**b**) channel 5, (**c**) channel 6, (**d**) channel 8, (**e**) channel 9. Part (**f**) is the corresponding RRMSEs.

**Figure 18:**
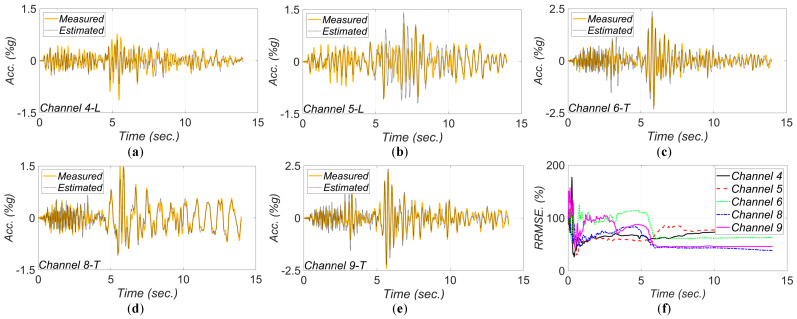
Comparison between measured and estimated (from updated model) responses at different measurement channels for 2004 IslaVista earthquake: (**a**) channel 4, (**b**) channel 5, (**c**) channel 6, (**d**) channel 8, (**e**) channel 9. Part (**f**) is the corresponding RRMSEs.

**Figure 19:**
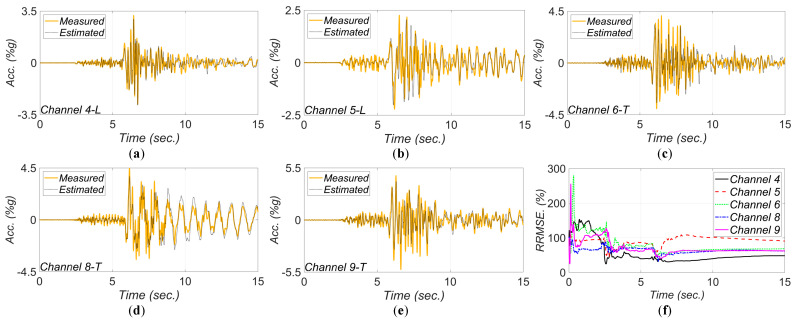
Comparison between measured and estimated (from updated model) responses at different measurement channels for 2013 IslaVista earthquake: (**a**) channel 4, (**b**) channel 5, (**c**) channel 6, (**d**) channel 8, (**e**) channel 9. Part (**f**) is the corresponding RRMSEs.

**Figure 20:**
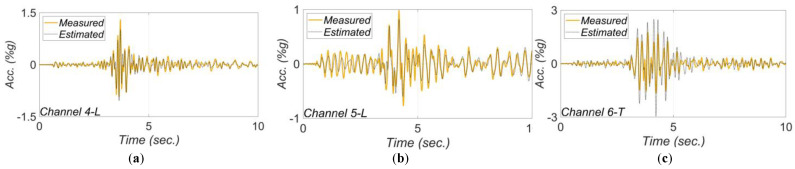

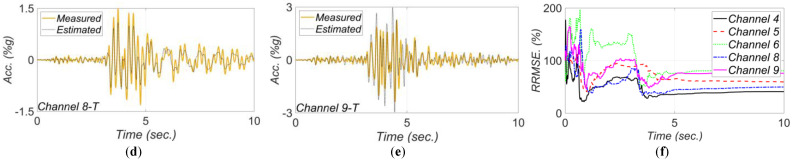
Comparison between measured and estimated (from updated model) responses at different measurement channels for 2017 Montecito earthquake: (**a**) channel 4, (**b**) channel 5, (**c**) channel 6, (**d**) channel 8, (**e**) channel 9. Part (**f**) is the corresponding RRMSEs.

**Figure 21:**
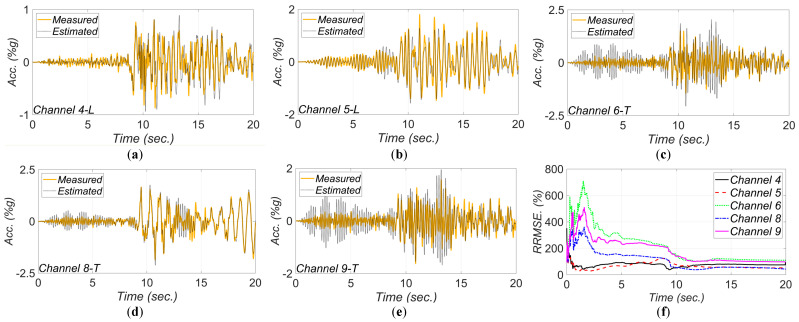
Comparison between measured and estimated (from updated model) responses at different measurement channels for 2018 Santa Cruz earthquake: (**a**) channel 4, (**b**) channel 5, (**c**) channel 6, (**d**) channel 8, (**e**) channel 9. Part (**f**) is the corresponding RRMSEs.

Figure 17, Figure 18, Figure 19, Figure 20 and Figure 21 show the comparison between the measured and estimated (from the updated models) acceleration responses. As seen, the predicted responses match well the measured responses in all events. However, as previously discussed in Section 3.3 and can be seen in the following figures, the accuracy in estimation of the responses is poor at the beginning of the earthquake event and improves in time.

## 5. Digital Twin and Virtual Sensing Application

**Figure 22:**
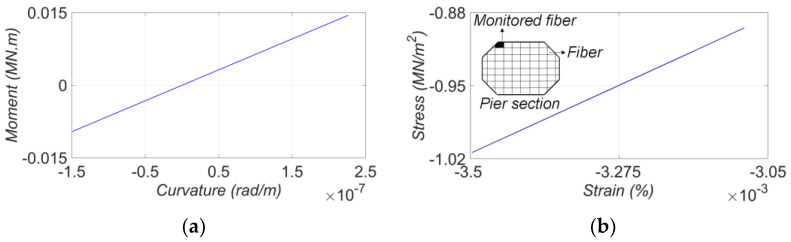
Local level response monitored in SRC bridge using its digital twin during 2013 IslaVista earthquake event: (**a**) moment-curvature response (about the transverse direction) at the lowest section of the east pier, and (**b**) estimated stress-strain response in the extreme fiber at the lowest section of the west pier.

The updated FE model is a replicate of the SRC bridge in the digital world given the training data, and is referred to as its digital twin (DT). The DT can be used to reconstruct local element- and material-level responses to diagnose damage at local levels of the bridge. For this purpose, the DT is used in a forward simulation for a given input motion and the local responses (e.g., section moment curvature, material stress-strain) are collected and used for damage localization and quantification. The process is often referred to as virtual sensing [45]. Although the level of available earthquake motions was small in this study and thus, no damage is expected in the structure, the application of the DT can still be demonstrated. It should be noted that the phrase “Digital Twin” may have different meanings and applications in different industries. Here, the digital twinning method is used for post-earthquake assessment of bridges. The data recorded in seismic events are considered as discontinuous real-time data assuming that no significant changes will happen between the events. Therefore, the DT will be updated after each seismic event and used for post-earthquake damage diagnosis. In this section, the SRC DT is established using the final estimates of the unknown model parameters from the 2013 IslaVista earthquake data (earthquake No. 3). Then, the estimated FIMs are used with the DT to monitor/estimate the structural responses. Figure 22 shows two examples of such monitored/estimated responses at local levels. Figure 22a presents the estimated moment-curvature response at the lowest section of the east pier about the transverse direction. Also, the predicted stress-strain response of the extreme concrete fiber at the lowest section of the west pier is shown in Figure 22b. As can be seen, the structure behaves in its linear-elastic regime during the 2013 IslaVista earthquake. However, a similar concept is applicable for stronger earthquakes to monitor the response nonlinearity at the local levels to infer potential damage location and extent.

## 6. Conclusions

This study presented the procedure for bridge digital twinning and virtual sensing through the application of an output-only time-domain model updating technique for post-earthquake damage diagnosis. In this technique the output-only seismic responses are integrated with the mechanics-based Finite Element (FE) model of a bridge through a Bayesian inference approach to jointly estimate the Foundation Input Motions (FIMs) and the unknown model parameters, and so the development of the bridge’s digital twin. The model updating is implemented using a sequential estimation window approach, in which the time domain is divided into sequential overlapping estimation windows. At each estimation window, the Probability Density Functions (PDF) of input and parameters are updated iteratively using an Unscented Kalman filtering method and transferred to the next estimation window. The technology solution for post-earthquake damage diagnosis will necessitate seamless implementation using parallel processing and high-performance computing (HPC) to enable a near real-time (e.g., within hours) processing capability.

The output-only time-domain model updating technique was first verified in a numerically simulated environment. For this purpose, a detailed FE model of the San Roque Canyon (SRC) bridge, located in CA, was developed in OpenSees. An identifiability analysis was performed to select the likely identifiable model parameters among 34 parameter candidates. Due to instrumentation sparsity and the low-amplitude seismic inputs, only a few (six) linear-related model parameters (concrete modulus of elasticity of deck, initial concrete modulus of elasticity of piers, stiffness of bearing pads, and Rayleigh damping coefficients) were found to be likely identifiable. The verification study presented good performance of the model updating technique in estimating the unknown model parameters jointly with the time history of FIMs.

In the next step, the model updating technique was repeated for five real-world earthquake data recorded at the SRC bridge station from 2003 to 2018. The estimated model parameters were compared between different earthquakes and the likely reasons for discrepancies were discussed. Estimates of the Rayleigh damping coefficients for different earthquakes revealed dependency of damping ratio on the intensity of the seismic excitation, i.e., a stronger earthquake with greater PGA resulted in a larger estimate of damping ratio. The estimates of bearing pad stiffness had large event-to-event variations, which were related to the level of intensity and potential modeling errors. Bearing pads were modeled as linear springs, while their stiffness often depends on the level and frequency content of the excitation. Furthermore, the estimated FIMs were compared with the Free-Field Motions (FFMs), and the discrepancies were discussed based on the expected soil-structure interaction effects. As expected, smaller discrepancies were observed between the FFMs and the estimated FIMs for the events with low frequency excitation. Furthermore, the measured responses of the bridge were compared with those estimated from the updated model (or digital twin). While the agreement between the measured and digital twin predicted responses was acceptable, a comparison of the relative root mean square errors between the numerically simulated case study and the real-world case studies clearly showed the inherent effects of modeling error in the FE model updating technique. Modeling errors refer to the mathematical idealization and simplifications in the model, which can result in biased and incorrect model updating outcomes. Finally, the digital twin was developed using the strongest studied earthquake. To demonstrate the virtual sensing application, the bridge’s digital twin was used to predict the local level response of the bridge given the strongest studied earthquake, an important capability that can be used for post-event damage diagnosis (damage detection, localization, and quantification). Although the levels of considered earthquakes were low in this study, the approach can be applied to large-scale and nonlinear models to diagnose damage as the result of material nonlinearity.

The objective of this study was to examine the application of the time domain output-only Bayesian FE model updating for damage identification and bridge digital twinning through a real-world case study. Further studies of this type with various structural systems (simple and complex, experimental, and real-world) and under various loading conditions are needed to better understand the limitation and capabilities of the Bayesian model updating approach as a technology solution for structural health monitoring and damage diagnosis.

## Figures and Tables

**Table 1:** Earthquake records considered in this study.

No.	Earthquake	Date	Distance (km)	PGA (g)	PSA in Transverse Direction (g)	PSA in Vertical Direction (g)	PSA in Longitudinal Direction (g)
1	San Simeon	22 December 2003	187.0	0.015	0.045	0.042	0.022
2	IslaVista	9 May 2004	27.2	0.016	0.026	0.047	0.013
3	IslaVista	29 May 2013	18.0	0.041	0.060	0.150	0.040
4	Montecito	23 April 2017	9.5	0.022	0.024	0.045	0.014
5	Santa Cruz	5 April 2018	67.9	0.016	0.021	0.058	0.019

**Table 2:** Candidate unknown model parameters. The model parameters are linked to their associated model components in Figure 6. The unknown parameters to be estimated using the model updating technique are highlighted in this table.

No.	Parameter	Description	Nominal Value
1	$E_{d}$	Elastic modulus of deck	27.8 GPa
2	$f_{c,c}^{’}$	Compressive strength of column	40.4 MPa
3	$E_{c}$	Initial elastic modulus of column	27.8 GPa
4	$k_{T}^{b}$	Transverse elastomeric shear stiffness of bearing pad	100 MN/m
5	$k_{L}^{b}$	Longitudinal elastomeric shear stiffness of bearing pad	10 MN/m
6	$m_{a}$	Embankment mass for abutment	53.0 kg
7	$k_{V}^{p}$	Vertical soil-foundation stiffness under pier	12.7 GN/m
8	$c_{V}^{p}$	Vertical soil-foundation damping coefficient under pier	240 MN.s/m
9	$k_{L}^{p}$	Longitudinal soil-foundation stiffness under pier	9.8 GN/m
10	$c_{L}^{p}$	Longitudinal soil-foundation damping coefficient under pier	220 MN.s/m
11	$k_{T}^{p}$	Transverse soil-foundation stiffness under pier	9.8 GN/m
12	$c_{T}^{p}$	Transverse soil-foundation damping coefficient under pier	190 MN.s/m
13	$k_{R,L}^{p}$	Rotational soil-foundation stiffness under pier about the longitudinal axis	170 GN.m/rad
14	$c_{R,L}^{p}$	Rotational soil-foundation damping coefficient under pier about the longitudinal axis	2.7GN.m.s/rad
15	$k_{R,T}^{p}$	Rotational soil-foundation stiffness under pier about the transverse axis	170 GN.m/rad
16	$c_{R,T}^{p}$	Rotational soil-foundation damping coefficient under pier about the transverse axis	2.7 GN.m.s/rad
17	$k_{R,V}^{p}$	Rotational soil-foundation stiffness under pier about the vertical axis	290 GN.m/rad
18	$c_{R,V}^{p}$	Rotational soil-foundation damping coefficient under pier about the vertical axis	3.6 GN.m.s/rad
19	$k_{L}^{a}$	Longitudinal soil-foundation stiffness under abutment	8.7 GN/m
20	$c_{L}^{a}$	Longitudinal soil-foundation damping coefficient under abutment	170 MN.s/m
21	$k_{T}^{a}$	Transverse soil-foundation stiffness under abutment	37 GN/m
22	$c_{T}^{a}$	Transverse soil-foundation damping coefficient under abutment	130 MN.s/m
23	$k_{V}^{a}$	Vertical soil-foundation stiffness under abutment	10 GN/m
24	$c_{V}^{a}$	Vertical soil-foundation damping coefficient under abutment	150 MN.s/m
25	$k_{R,L}^{a}$	Rotational soil-foundation stiffness under abutment about its longitudinal axis	300 GN.m/rad
26	$c_{R,L}^{a}$	Rotational soil-foundation damping coefficient under abutment about the longitudinal axis	4.7 GN.m.s/rad
27	$k_{R,V}^{a}$	Rotational soil-foundation stiffness under abutment about the vertical axis	240 GN.m/rad
28	$c_{R,V}^{a}$	Rotational soil-foundation damping coefficient under abutment about the vertical axis	390 GN.m.s/rad
29	$k_{L}^{fs}$	Far-field soil-embankment stiffness in longitudinal direction	8.7 GN/m
30	$c_{R}^{fs}$	Far-field soil-embankment radiation damping coefficient in the longitudinal direction	38 MN.s/m
31	$c_{L}^{fs,m}$	Far-field soil-embankment material damping coefficient in the longitudinal direction	140 MN.s/m
32	$k_{L}^{bs}$	Soil-backwall initial stiffness in the longitudinal direction	$10^{5}\;\mathrm{GN}/m$
33	$a_{0}$	Mass proportional Rayleigh damping coefficient	$0.6\;s^{-1}$
34	$a_{1}$	Stiffness proportional Rayleigh damping coefficient	0.003 s

## Data Availability

Data and materials supporting the results or analyses presented in this work are available upon reasonable request from the corresponding author.
